# Facility-based and home-based multidomain interventions including cognitive training, exercise, diet, vascular risk management, and motivation for older adults: a randomized controlled feasibility trial

**DOI:** 10.18632/aging.203213

**Published:** 2021-06-18

**Authors:** So Young Moon, Chang Hyung Hong, Jee Hyang Jeong, Yoo Kyoung Park, Hae Ri Na, Hong-Sun Song, Byeong C. Kim, Kyung Won Park, Hee Kyung Park, Muncheong Choi, Sun Min Lee, Buong-O Chun, Seong-Ho Koh, Sun Ah Park, Hyun-Hee Park, Jeong-Hwa Jin, Eun-Hye Lee, Sue Min Kim, Song Mi Han, Jun Seok Kim, Jungsoon Ha, Seong Hye Choi

**Affiliations:** 1 Department of Neurology, Ajou University School of Medicine, Suwon 16499, Republic of Korea; 2 Department of Psychiatry, Ajou University School of Medicine, Suwon 16499, Republic of Korea; 3 Department of Neurology, Ewha Womans University School of Medicine, Seoul 07804, Republic of Korea; 4 Department of Medical Nutrition, Graduate School of East-West Medical Nutrition, Kyung Hee University, Yongin 17104, Republic of Korea; 5 Department of Neurology, Bobath Memorial Hospital, Seongnam 13552, Republic of Korea; 6 Department of Sports Sciences, Korea Institute of Sports Science, Seoul 01794, Republic of Korea; 7 Department of Neurology, Chonnam National University Medical School, Gwangju 61469, Republic of Korea; 8 Department of Neurology, Dong-A University College of Medicine, Busan 49201, Republic of Korea; 9 Department of Physical Education, Kookmin University, Seoul 02707, Republic of Korea; 10 Department of Neurology, Hanyang University College of Medicine, Seoul 04763, Republic of Korea; 11 Department of Translational Medicine, Hanyang University Graduate School of Biomedical Science and Engineering, Seoul 04763, Republic of Korea; 12 Lab for Neurodegenerative Dementia, Department of Anatomy, Ajou University School of Medicine, Suwon 16499, Republic of Korea; 13 Neuroscience Graduate Program, Department of Biomedical Sciences, Ajou University Graduate School of Medicine, Suwon 16499, Republic of Korea; 14 Department of Biomedical Systems Engineering, Korea Polytechnics University, Gyeonggi 13122, Republic of Korea; 15 Department of Neurology, Inha University School of Medicine, Incheon 22332, Republic of Korea

**Keywords:** dementia, prevention, lifestyle, feasibility, randomized controlled trial

## Abstract

We aimed to evaluate the feasibility of multidomain intervention (MI) tailored to the Korean context. In an outcome assessor-blinded, randomized controlled trial, participants without dementia and with one or more modifiable dementia risk factors, aged 60-79 years, were randomly assigned to the facility-based MI (FMI; n=51), the home-based MI (HMI; n=51), or the control group receiving general health advice (n=50). The 24-week intervention comprised vascular risk management, cognitive training, social activity, physical exercise, nutrition guidance, and motivational enhancement. The FMI participants performed all intervention programs at a facility three times a week. The HMI participants performed some programs at a facility once every 1-2 weeks and performed others at home. The primary outcome was feasibility measured through retention, adherence, and at least no differences from the control group in the Repeatable Battery for the Assessment of Neuropsychological Status (RBANS). In the FMI and HMI groups, the retention rates were 88.2% and 96.1%, and adherence to the intervention was 94.5% and 96.8%, respectively. The RBANS total scale index score improved significantly in the FMI (5.46 ± 7.50, *P* = 0.004) and HMI (5.50 ± 8.14, *P* = 0.004) groups compared to the control group (-0.74 ± 11.51). The FMI and HMI are feasible and there are indicators of efficacy.

## INTRODUCTION

As life expectancy increases worldwide, dementia has rapidly become a huge public health problem. A recent meta-analysis of population-based observational studies revealed that modifying risk factors may prevent or delay dementia by up to 40% [1]. Additionally, observational cohort studies have revealed that decreases in the age-adjusted incidences of dementia in some Western countries may be due to changes in lifestyle-related risk factor profiles, such as improved management of vascular risk factors or increased educational opportunities [2, 3].

The Finnish Geriatric Intervention Study to Prevent Cognitive Impairment and Disability (FINGER), a two-year randomized controlled trial (RCT), demonstrated that it is possible to improve the cognitive function of older adults at risk of developing dementia through multidomain lifestyle interventions, including dietary counseling, physical exercise, cognitive training, and vascular and metabolic risk monitoring [4]. However, two other RCTs (the Multidomain Alzheimer Preventive Trial [5] and Prevention of Dementia by Intensive Vascular Care trial [6]) failed to find similar effects on cognitive improvement or the prevention of dementia. Therefore, further evidence is necessary so that public health recommendations can encourage people to adopt lifestyle interventions that prevent dementia.

The World Health Organization emphasized the importance of further research regarding the feasibility and efficacy of multidomain interventions, adjusted to specific geographical and cultural contexts [7]. The World-Wide FINGERS (WW-FINGERS) network was established in 2017 to support global multidomain dementia prevention trials and share experiences and data [8]. The SoUth Korean study to PrEvent cognitive impaiRment and protect BRAIN health through lifestyle intervention in at-risk elderly people (SUPERBRAIN) reported in this paper is also part of the WW-FINGERS network [8].

South Korea is expected to become a superaged society by 2025 [9]. Therefore, it is essential to relieve the burden of dementia through its prevention. To prevent and manage dementia, the Korean government recently instigated a community-based welfare policy to build public dementia centers in approximately 250 regions across the country. Effective dementia prevention programs that can be implemented in these facilities are urgently required. In addition, there is a limit to the number of people who can receive intensive multidomain interventions at these facilities; therefore, an effective home-based program is essential to reach more elderly people in need. We developed a facility-based multidomain intervention (FMI) program and a home-based multidomain intervention (HMI) program suitable for older Koreans [9].

In the SUPERBRAIN, we aimed to assess the feasibility of the FMI and HMI programs in at-risk older Koreans. The intervention programs are deemed feasible when the following criteria are met: participant retention rate of at least 75% [10], adherence rate to the intervention of at least 75%, and at least no differences from the control group in the primary cognitive outcome analysis (i.e., no negative effects) [11]. Feasibility was determined separately for the FMI and HMI.

## RESULTS

### Retention rate and adherence

**Figure 1:**
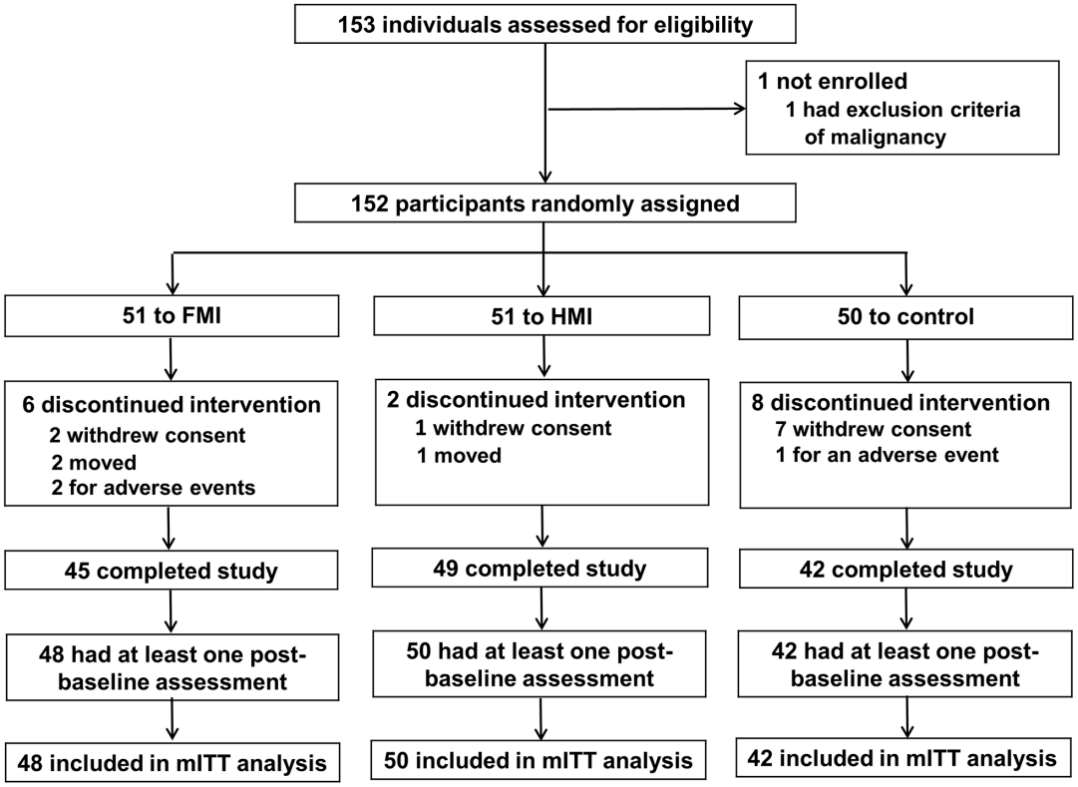
**Trial profile.** FMI: facility-based multidomain intervention; HMI: home-based multidomain intervention; mITT: modified intention-to-treat.

Between May 29 and August 20, 2019, 152 participants were randomly assigned to three groups: 51 to the FMI group, 51 to the HMI group, and 50 to the control group (Figure 1). The study ended in February 2020. Finally, 45, 49, and 42 participants completed the study in the FMI, HMI, and control groups, respectively. The retention rates at week 24 were 88.2%, 96.1%, and 84.0% in the FMI, HMI, and control groups, respectively. Differences in the retention rates of the groups were not significant (*P* = 0.13). The modified intention-to-treat (mITT) analysis participants were 48, 50, and 42 in the FMI, HMI, and control groups, respectively.

There were no significant differences in any demographic or clinical characteristics between the control and intervention groups in the mITT population (Table 1) or among the randomized participants (Supplementary Table 1). At baseline, there were no significant differences in age, sex, education, or scores on the Mini-Mental State Examination (MMSE) [12], Geriatric Depression Scale-15 items (GDS-15) [13], Clinical Dementia Rating-Sum of Boxes (CDR-SB) [14], Bayer-Activities of Daily Livings (ADL) [15], and Repeatable Battery for the Assessment of Neuropsychological Status (RBANS) [16] between the mITT population and those excluded from the mITT analysis.

**Table 1:** Baseline characteristics of the modified ITT population.

	**FMI group (n = 48)**	**HMI group (n = 50)**	**Control group (n = 42)**
**Demographic characteristics**			
Age at baseline visit, years	71.6 (4.8)	70.9 (5.0)	70.1 (4.6)
Number of women	35 (72.9%)	36 (72.0%)	33 (78.6%)
Education, years	9.8 (4.7)	10.1 (5.0)	10.3 (4.7)
**Medical history**			
Hypertension	23 (47.9%)	27 (54.0%)	25 (59.5%)
Diabetes Mellitus	9 (18.8%)	13 (26.0%)	14 (33.3%)
Dyslipidemia	29 (60.4%)	32 (64.0%)	30 (71.4%)
Cardiac disease	3 (6.3%)	2 (4.0%)	2 (4.8%)
History of stroke	3 (6.3%)	3 (6.0%)	5 (11.9%)
Mild cognitive impairment	17 (35.4%)	13 (26.0%)	9 (21.4%)
**Vascular factors**			
Systolic blood pressure, mmHg	127.8 (16.1)	126.7 (13.0)	132.1 (16.6)
Diastolic blood pressure, mmHg	73.8 (10.4)	74.3 (10.2)	74.8 (8.3)
Total cholesterol, mg/dL	184.9 (39.7)	190.6 (35.5)	174.3 (41.2)
LDL-cholesterol, mg/dL	103.9 (37.5)	111.7 (32.9)	95.8 (32.8)
Triglyceride, mg/dL	139.5 (72.9)	143.1 (78.6)	154.0 (117.9)
HDL-cholesterol, mg/dL	53.9 (12.6)	53.8 (14.7)	52.5 (13.5)
Fating plasma glucose, mg/dL	102.7 (29.9)	110.2 (33.7)	110.0 (39.9)
Body Mass Index, kg/m^2^	23.8 (2.1)	24.3 (3.1)	25.1 (2.8)
Abdominal circumference, cm	82.7 (7.1)	84.5 (8.9)	85.6 (8.4)
**Lifestyle factors**			
Current smokers	2 (4.2%)	1 (2.0%)	1 (2.4%)
At-risk alcohol drinking^†^	7 (14.6%)	6 (12.0%)	1 (2.4%)
Physical activity, MET x min per week	2160 (1787)	2820 (4628)	2203 (2693)
**Cognition**			
Mini-Mental State Examination	28.1 (1.8)	28.0 (1.8)	27.2 (2.5)
RBANS total scale index score	100.7 (19.3)	101.5 (16.3)	101.0 (19.9)
Clinical Dementia Rating-Sum of Boxes	0.46 (0.56)	0.49 (0.60)	038 (0.43)
Geriatric Depression Scale-15 items	4.5 (3.8)	4.2 (4.2)	4.2 (3.4)

Values are shown as the mean (SD) or number (%). ITT, intention-to-treat; FMI, facility-based multidomain intervention; HMI, home-based multidomain intervention; LDL, low-density lipoprotein; HDL, high-density lipoprotein; MET, metabolic equivalents; RBANS, Repeatable Battery for the Assessment of Neuropsychological Status. *Chi-square test for categorical variables and analysis of variance for continuous variables. †Four drinks or more during a day, or more than seven drinks per week.

**Figure 2:**
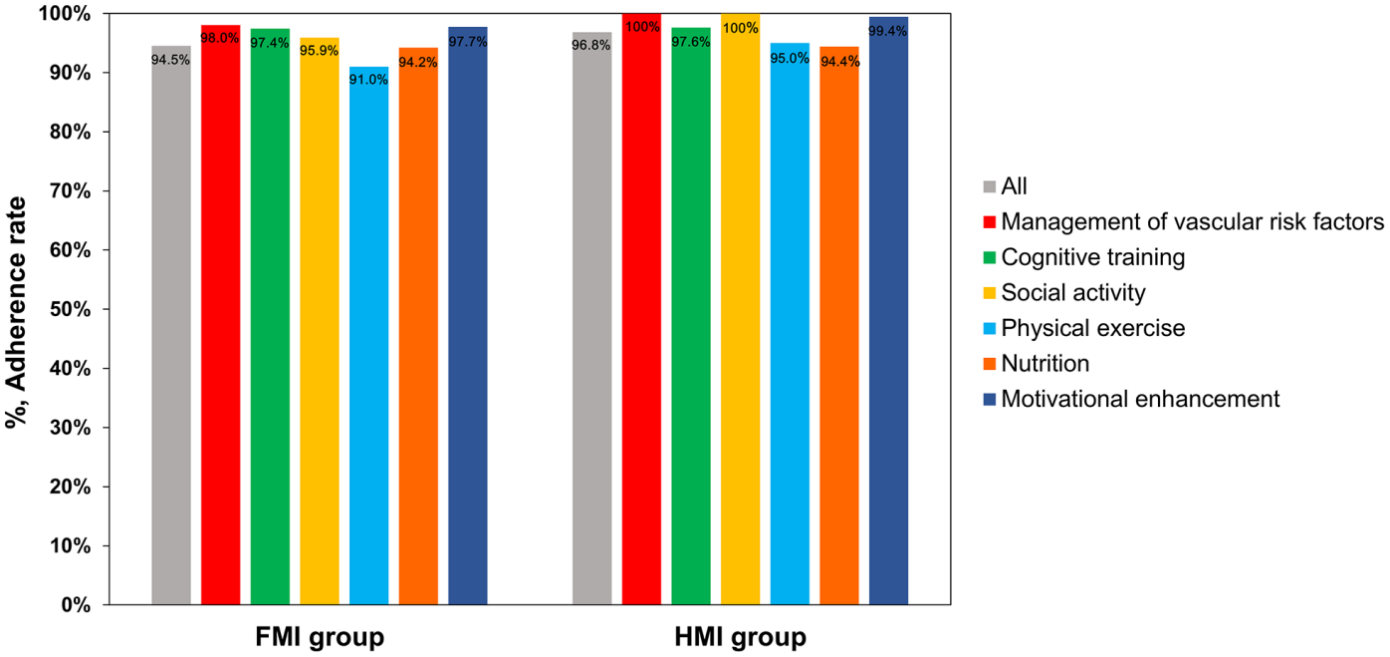
**The adherence rates in the FMI and HMI groups.** FMI: facility-based multidomain intervention; HMI: home-based multidomain intervention.

The total adherence rates were 94.5% (95% confidence interval [CI], 91.4-97.6%) in the FMI group and 96.8% (95% CI, 95.4-98.1%) in the HMI group. The adherence rates to the vascular and metabolic risk factor management program, cognitive training program using a tablet-based application or workbooks, social activity program, physical exercise program, nutritional program, and motivational enhancement program were 98.0%, 97.4%, 95.9%, 91.0%, 94.2%, and 97.7% in the FMI group, and 100%, 97.6%, 100%, 95.0%, 94.4%, and 99.4% in the HMI group (Figure 2).

### RBANS

**Figure 3:**
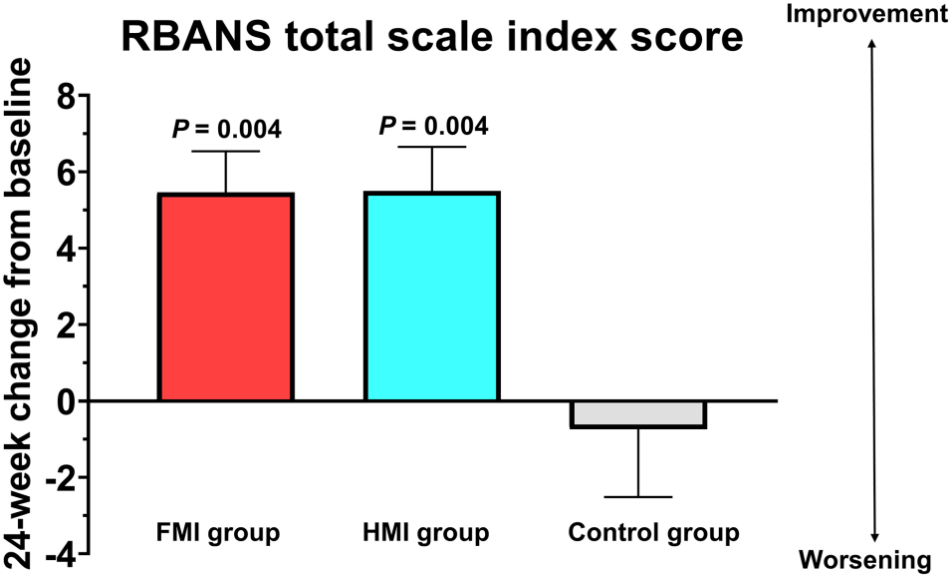
**Mean changes from baseline at study end in RBANS total scale index score.** The RBANS total scale index score significantly improved by an average of 5.46 (SD = 7.50) points in the FMI group and an average of 5.50 (8.14) points in the HMI group compared to the control group with a decline by an average of 0.74 (11.51) points. Bars and lines show the mean and standard error of the mean, respectively. The *P* values represent the results of the comparison between each intervention group and the control group by the analysis of covariance with the baseline level as a covariate and Bonferroni correction. FMI, facility-based multidomain intervention; HMI, home-based multidomain intervention.

The RBANS total scale index score significantly improved by an average of 5.46 (SD = 7.50, *P* = 0.004) points in the FMI group and an average of 5.50 (SD = 8.14, *P* = 0.004) points in the HMI group compared to the control group with a decline by an average of 0.74 (SD = 11.51) points, at the end of the study (Figure 3). The Cohen’s *d* to represent intervention effect size was 0.64 and 0.63 for each FMI and HMI group. When comparing the index score for each cognitive domain of the RBANS with the control group, visuoconstruction ability in each intervention group, delayed memory in the FMI group, and attention in the HMI group were significantly improved (Table 2). Cognitive training using a tablet-based application was conducted with 67 participants, and 35 received cognitive training using workbooks. Participants using workbooks for cognitive training were older and more likely to be female than those using the cognitive training application. The change from baseline to the study endpoint in the RBANS total scale index score did not differ between participants using the cognitive training application and those using the workbooks after adjusting for their age, gender, and baseline scores (6.11 ± 8.09 vs. 4.24 ± 7.13, *P* = 0.28).

**Table 2:** Mean changes in the index scores of cognitive domains of the RBANS.

**Index score**	**Baseline scores**				**Changes from baseline to study end**				P*	
	**FMI (n = 48)**	**HMI (n = 50)**	**Control (n = 42)**		**FMI (n = 48)**	**HMI (n = 50)**	**Control (n = 42)**		**FMI vs. Control**	**HMI vs. Control**
Immediate memory	101.3 (13.9)	99.3 (14.7)	100.7 (15.4)		5.33 (9.65)	3.56 (12.53)	2.02 (10.85)		0.11	0.62
Visuoconstruction	93.3 (16.7)	94.5 (15.5)	91.5 (16.0)		0.75 (14.00)	0.90 (14.45)	-6.36 (14.20)		**0.01**	**0.003**
Language	104.0 (15.8)	108.2 (13.3)	106.3 (13.9)		1.60 (12.50)	1.98 (12.59)	-0.67 (12.02)		0.57	0.12
Attention	104.3 (17.9)	103.2 (16.8)	101.8 (18.2)		0.13 (8.34)	2.46 (9.46)	-2.05 (12.13)		0.19	**0.02**
Delayed memory	94.3 (16.1)	94.6 (14.9)	98.2 (17.2)		10.08 (10.16)	9.06 (12.03)	4.43 (10.20)		**0.02**	0.11

Values are shown as the mean (SD). FMI, facility-based multidomain intervention; HMI, home-based multidomain intervention; RBANS, Repeatable Battery for the Assessment of Neuropsychological Status. Higher scores indicate better performance in all index scores. *Analysis of covariance with each baseline score as a covariate.

### Secondary outcomes

Compared to the control group, depression, Quality of Life (QOL), control of vascular risk factors such as blood Pressure (BP) and body fat mass, dietary habits evaluated by Nutrition Quotient for the Elderly (NQ-E) [17], physical performance evaluated by Timed UP and Go (TUG) test, and motivation evaluated by the Self Determination Index (SDI) [18] were significantly improved in both the FMI and HMI groups (Table 3). Additionally, sleep quality, subjective memory impairment evaluated by the Prospective and Retrospective Memory Questionnaire (PRMQ) [19], and the Short Physical Performance Battery (SPPB) score [20] were significantly improved in the HMI group compared to the control group. Changes in additional vascular and nutritional factors and changes in the remainder of Korean National Physical Fitness Evaluation Program (K-NPFEP) are presented in Supplementary Table 2.

**Table 3:** Mean changes in the main secondary outcome measures from baseline to the study endpoint.

	**Baseline scores**				**Changes from baseline to study end**				P^*^	
	**FMI (n = 48)**	**HMI (n = 50)**	**Control (n = 42)**		**FMI (n = 48)**	**HMI (n = 50)**	**Control (n = 42)**		**FMI vs. Control**	**HMI vs. Control**
Mini-Mental State Examination^†^	28.1 (1.8)	28.0 (1.8)	27.2 (2.5)		0.04 (1.92)	0.18 (1.79)	-0.24 (2.94)		0.37	0.28
CDR-SB	0.46 (0.56)	0.49 (0.60)	0.38 (0.43)		0.03 (0.53)	-0.09 (0.46)	0.50 (2.50)		0.20	0.10
Prospective Memory Test^†^	9.1 (2.6)	8.8 (2.7)	9.3 (2.6)		0.24 (2.56)	0.48 (2.98)	0.15 (2.71)		0.99	0.89
PRMQ	34.3 (10.6)	34.0 (11.2)	32.0 (11.6)		-2.46 (9.87)	-3.72 (9.98)	0.90 (6.79)		0.11	**0.01**
PRMQ by caregiver	31.1 (10.5)	29.1 (9.3)	26.4 (10.3)		-3.58 (8.49)	-2.12 (8.16)	2.17 (13.74)		0.16	0.19
Cognitive Complaint Interview	4.4 (2.0)	4.2 (2.1)	3.8 (2.4)		-1.31 (2.20)	-1.12 (1.75)	-0.61 (1.91)		0.28	0.24
Geriatric Depression Scale-15 items	4.5 (3.8)	4.2 (4.2)	4.2 (3.4)		-1.35 (3.45)	-1.24 (3.09)	0.12 (2.60)		**0.03**	**0.01**
Bayer Activities of Daily Living	1.76 (1.11)	1.51 (0.63)	1.64 (1.02)		-0.30 (0.90)	-0.13 (0.66)	0.11 (1.12)		0.06	0.14
Quality of Life in Alzheimer’s disease^†^	33.8 (5.6)	33.2 (5.3)	32.5 (6.1)		2.03 (5.27)	3.01 (4.03)	0.74 (4.49)		**0.04**	**0.001**
Pittsburgh Sleep Quality Index	6.5 (3.7)	7.2 (3.6)	7.4 (4.1)		0.06 (3.69)	-0.76 (3.54)	0.51 (3.30)		0.24	**0.04**
SPPB^†^	11.4 (1.2)	11.3 (1.2)	11.1 (1.3)		0.33 (1.17)	0.52 (1.20)	0.33 (1.37)		0.24	**0.03**
Timed Up and Go test, sec	6.5 (1.6)	6.5 (1.5)	6.6 (1.8)		-0.18 (1.84)	-0.17 (1.47)	0.54 (2.46)		**0.02**	**0.01**
Mini Nutritional Assessment^†^	12.0 (2.1)	11.9 (2.1)	12.1 (2.3)		0.56 (2.24)	0.78 (2.48)	-0.02 (2.40)		0.27	0.14
Nutrition Quotient for elderly^†^	64.5 (10.5)	64.9 (9.7)	64.9 (9.4)		5.67 (8.93)	6.00 (7.77)	1.41 (8.12)		**0.01**	**0.003**
Systolic blood pressure, mmHg	127.8 (16.1)	126.7 (13.0)	132.1 (16.6)		-1.54 (18.49)	0.02 (14.96)	0.44 (17.12)		**0.03**	0.08
Diastolic blood pressure, mmHg	73.8 (10.4)	74.3 (10.2)	74.8 (8.3)		-1.54 (12.53)	-4.06 (10.26)	0.68 (8.77)		0.13	**0.006**
Body fat mass, kg	18.6 (4.9)	19.4 (5.6)	20.9 (4.8)		-0.19 (1.67)	-0.22 (2.11)	0.68 (2.41)		**0.009**	**0.02**
Self Determination Index^†^	20.7 (16.1)	17.6 (21.5)	15.0 (23.1)		10.13 (17.12)	14.68 (21.71)	0.63 (14.95)		**0.001**	**<0.001**

Values are shown as the mean (SD). FMI, facility-based multidomain intervention; HMI, home-based multidomain intervention; CDR-SB, Clinical Dementia Rating-Sum of Boxes; PRMQ, Prospective Retrospective Memory Questionnaire; SPPB, Short Physical Performance Battery. *Analysis of covariance with each baseline score as a covariate †Higher scores indicate better performance.

No significant differences were observed between the FMI group and the HMI group in retention rate, adherence rate, and changes in the RBANS total scale index score and secondary outcomes.

### Exploratory blood biomarkers

**Figure 4:**
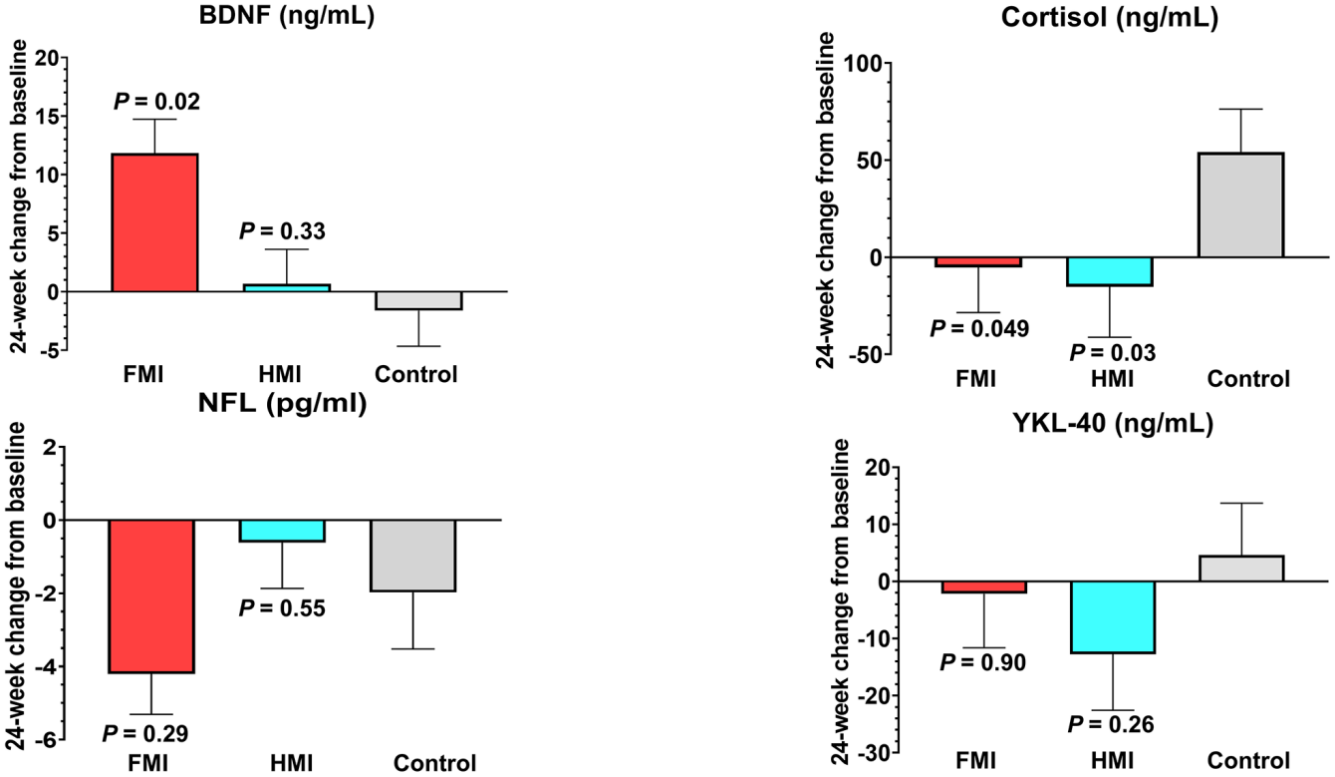
**Mean changes from baseline at study end of exploratory blood biomarkers.** Compared to the control group (-1.62 ± 19.01 ng/mL), serum brain-derived neurotrophic factor (BDNF) levels were significantly increased in the FMI group (11.83 ± 20.06 ng/mL) but not in the HMI group (0.67 ± 20.61 ng/mL). Compared to the control group (54.18 ± 136.01 ng/mL), plasma cortisol levels were significantly decreased in both the FMI (-5.29 ± 154.01 ng/mL) and HMI groups (-15.29 ± 172.09 ng/mL) at the study endpoint. The mean changes in serum neurofilament light chain (NFL) levels from baseline to the study endpoint were as follows: 4.21 (7.65) pg/ml decrease in the FMI group, 0.62 (8.74) pg/ml decrease in the HMI group, and 1.98 (9.77) pg/ml decrease in the control group. The mean changes in serum YKL-40 levels at the study endpoint from baseline were as follows: 2.19 (65.33) ng/mL decrease in the FMI group, 12.76 (68.58) ng/mL decrease in the HMI group, and 4.62 (57.49) ng/mL increase in the control group. Bars and lines show the mean and standard error of the mean, respectively. The *P* values represent the results of comparisons between each intervention group and the control group by the analysis of covariance with the baseline level as a covariate. FMI, facility-based multidomain intervention; HMI, home-based multidomain intervention.

Compared to the control group (54.18 ± 136.01 ng/mL), plasma cortisol levels were significantly decreased in both the FMI (-5.29 ± 154.01 ng/mL, *P* = 0.049) and HMI groups (-15.29 ± 172.09 ng/mL, *P* = 0.03) at the study endpoint (Figure 4 and Supplementary Table 2). Serum brain-derived neurotrophic factor (BDNF) levels were significantly increased in the FMI group compared to the control group (11.83 ± 20.06 ng/mL vs. -1.62 ± 19.01 ng/mL, *P* = 0.02). Serum chitinase-3-like protein 1 (YKL-40) levels decreased in the FMI and HMI groups and increased in the control group. Although not statistically significant, serum neurofilament light chain (NFL) levels decreased more in the FMI group than in the control group.

### Safety

The safety analysis population was 152. Table 4 summarizes the adverse events (AEs) that occurred in three or more participants. The incidence of participants reporting at least one AE during the study period was 33.3%, 39.2%, and 24.0% in the FMI, HMI, and control groups, respectively (*P* = 0.26). There were no AEs with significant differences in incidence among the groups. Three cases of intervention-related AEs were reported: shoulder pain in the FMI group and knee pain and abdominal pain in the HMI group. Five serious AEs (SAEs) were reported. The SAEs occurring in the FMI group included rotator cuff tear surgery and obstructive prostate hypertrophy. The SAEs occurring in the control group included acute cerebral infarction, general weakness, and spinal compression fracture. None of the SAEs were reported to be related to the study.

**Table 4:** Adverse events reported during the study.

	**FMI group**	**HMI group**	**Control group**	P^*^
Any adverse event	17 (33.3%)	20 (39.2%)	12 (24.0%)	0.26
Upper respiratory infection	6 (11.8%)	4 (7.8%)	5 (10.0%)	0.80
Musculoskeletal pain	3 (5.9%)	5 (9.8%)	2 (4.0%)	0.49
Dizziness	2 (3.9%)	2 (3.9%)	2 (4.0%)	1.00
Contusion	4 (7.8%)	1 (2.0%)	0 (0.0%)	0.07
Dyspepsia	2 (3.9%)	2 (3.9%)	1 (2.0%)	0.82
Fracture	1 (2.0%)	1 (2.0%)	2 (4.0%)	0.76
Diarrhea	2 (3.9%)	2 (3.9%)	0 (0.0%)	0.37
Ligament injury	2 (3.9%)	1 (2.0%)	1 (2.0%)	0.78
Fall	2 (3.9%)	1 (2.0%)	0 (0.0%)	0.37

Values are shown as numbers (%). FMI, facility-based multidomain intervention; HMI, home-based multidomain intervention. *Chi-square test.

## DISCUSSION

The total adherence rates of the FMI and HMI groups were 94.5% and 96.8%, respectively, which were over 75%. The retention rates of the FMI and HMI groups were 88.2% and 96.1%, respectively, which were also over 75%. The RBANS total scale index score significantly improved in both the FMI and HMI groups compared to the control group. Therefore, both the FMI and HMI programs of the SUPERBRAIN exhibit excellent feasibility. Furthermore, the intervention programs proved to be relatively safe.

The adherence rates in this study were higher than those in previous studies [21]. The reasons for this high adherence may be due to several factors. First, the SUPERBRAIN program was distinguished from other multidomain lifestyle intervention programs in that it included a motivational enhancement program [9]. The purpose of the motivational enhancement program was to act as a psychological resource that helps maintain the performance of dementia prevention activities by inducing, maintaining, and strengthening motivation. [22, 23]. Throughout the whole intervention period, participants were encouraged to sustain their motivation by group counseling sessions, the family as a coach (FAMICO) program, and self-assessment regarding participants’ dementia prevention activities. In the group counseling session conducted by psychologists, participants were educated regarding the importance of lifestyle changes for the prevention of dementia. In the FAMICO program, family members participated in encouraging participants by creating cheering video messages that were delivered weekly to participants through tablet-based applications or mobile phones. In addition, participants performed weekly self-assessments of dementia prevention activities. As a result, in the current trial, a significant improvement in the SDI scores for measuring motivation was revealed in both the FMI and HMI groups at the study endpoint. Second, to monitor adherence, we used a digital platform and wearables, as well as face-to-face assessments. The study coordinators monitored the participants’ homework thoroughly, both remotely and face-to-face; they provided feedback to the HMI participants on the phone and face-to-face, and they motivated participants to maintain the intervention program at home. In particular, tablet-based cognitive and motivational applications were configured to allow administrators to view all participants’ data and to check their progress on the administration homepage, which made it possible to monitor intervention adherence using objective measures. In addition, we used the Fitbit for the home-based exercise intervention to check whether the participants actually performed the exercises according to the protocol. This is a better approach rather than relying solely on their self-report. Third, participants in the FMI group visited a facility three times a week to participate in supervised intervention programs; these visitations may have resulted in high adherence. Fourth, participants performed various activities. Therefore, it was not boring. They enjoyed the program, which may have resulted in high adherence. Fifth, the intervention period in this study lasted 6 months, so the adherence and retention rates may have been higher than in the 2- or 3-year programs.

In this study, the HMI group showed remarkable results comparable to those of the FMI group. The improvement in RBANS scores in the HMI group was similar to that of the FMI group. The retention and adherence rates were slightly higher among the HMI participants than among the FMI participants. In the FINGER, adherence was highest for cardiovascular monitoring and nutritional counseling and lowest for unsupervised computer-based cognitive training [21]. When we planned this trial, we were concerned that HMI participants would have low adherence to cognitive training and exercise at home [9]. Therefore, much effort has been made to maintain the adherence of HMI participants through reinforcement of motivation and thorough feedback based on monitoring intervention adherence using objective measures. Since HMI participants were able to perform the program at home, they could easily adjust their schedule at any time to suit their situation if their motivation could be sustained [24]. The HMI program may prove to be more economical due to reduced labor and a lack of venue costs over time. Given that this study was conducted prior to the COVID-19 pandemic, FMI may be less feasible in the future due to the COVID-19 pandemic. In particular, older people may be at risk for CVOID-19 through group intervention sessions even if they are vaccinated. The HMI program may be useful in the current global environment since outdoor activities are limited during the international COVID-19 quarantine or during episodes of high air pollution. The HMI program of SUPERBRAIN is a blended model of face-to-face and non-face-to-face programs, so participants retain their interest and are motivated continuously.

The RBANS total scale index score, as the primary efficacy outcome, significantly improved in both the FMI and HMI groups compared to the control group. The group sizes achieved 83.6% and 82.9% power in the FMI and HMI groups, respectively, to evaluate efficacy. The effect sizes of FMI and HMI are medium considering the Cohen’s *d* scores [25]. In addition, although the results regarding secondary outcomes should be interpreted with much caution, each intervention component-related measures improved in the FMI and HMI groups. The multidomain lifestyle intervention also beneficially affected depression, sleep quality, and QOL. Furthermore, plasma cortisol levels were significantly decreased in both the FMI and HMI groups at the end of the study. Serum BDNF levels were significantly increased in the FMI group compared to the control group. Therefore, it is possible that the multidomain intervention may be effective in improving cognitive function through inactivation of the hypothalamic-pituitary-adrenal (HPA) axis [26, 27] and enhance brain plasticity [28, 29].

There are some limitations in this study. First, it is a single-blinded study, not double-blinded, accomplished through blinding the outcome assessor. Although participant groups were not exposed to the outcome assessor, it is possible that complete blindness was not achieved. The study coordinators who evaluated adherence were not blinded to which participants were in the intervention groups. Second, this study was a feasibility RCT. Further verification through a large-scale RCT powered for efficacy is necessary. Third, we should accept that the Fitbit was not able to monitor the activity during certain exercises, such as finger-and-toe exercises and strength exercises in a sitting position. Therefore, with the Fitbit, there was a limit to monitoring participants’ performance or exercise intensity during the entire exercise period. For the purpose of the further exact monitoring of adherence to home-based exercises, including nonaerobic exercise, measures other than the Fitbit, such as real-time transmission of video streaming over communication networks, should be considered. Fourth, the group allocation was actively disclosed to the study participants, and the control group was aware that a multidomain intervention was available, but they could access it only after the study ended. The decline in both cognition and depressive symptoms in the control group could have been partially affected by this element.

In conclusion, both the FMI and HMI programs of the SUPERBRAIN are feasible and likely to prevent cognitive impairment in at-risk older people. Multidomain lifestyle interventions may influence the brain through inactivation of the HPA axis and enhanced brain plasticity. Further verification through a large-scale RCT is required in the future.

## MATERIALS AND METHODS

### Study design and participants

This study was a 24-week, multicenter, outcome assessor-blinded RCT with a multi-arm parallel design conducted in three hospitals and five public health centers across South Korea. The study protocol has previously been published [9]. Participants were recruited among older adults who visited outpatient clinics or public health centers for memory complaints.

The inclusion criteria were as follows: aged between 60 and 79 years; having at least one modifiable dementia risk factor such as hypertension [30], diabetes mellitus (DM) [31], dyslipidemia [32], smoking [33], obesity [34], abdominal obesity [35], metabolic syndrome [36], educational level of ≤ 9 years, social isolation [9], and physical inactivity [9]; a MMSE [12] Z score of ≥ -1.5; able to perform independent ADL; being able to pass a literacy test [37]; and having a reliable informant who can provide investigators with the requested information. The exclusion criteria were major psychiatric illness; overt dementia; other neurodegenerative diseases; malignancy within the last 5 years; cardiac stent or revascularization within the previous year; serious or unstable symptomatic cardiovascular diseases; other serious or unstable medical diseases; any conditions preventing cooperation with the interventions, as determined by a study doctor; a significant laboratory abnormality that may result in cognitive impairment; inability to safely participate in the exercise program; and simultaneous participation in any other intervention trial.

The study was conducted in accordance with the International Conference on Harmonization Good Clinical Practices Guidelines [38]. Institutional review boards approved the protocol and consent forms at each institution before the study began. Written informed consent was obtained from all potential participants before their enrollment. This trial was registered with ClinicalTrials.gov (NCT03980392).

### Randomization

In a 1:1:1 ratio, participants were randomly assigned to the FMI, HMI, and control groups at baseline. Randomization was achieved through a permuted block randomization method, with block sizes of three and six, through SAS macro programming, and was stratified by the participating center. The allocation sequence was known only to the independent statistical specialist. Cognitive outcome assessors remained blind to the assigned groups; they were not involved in the intervention activities. Participants were instructed not to discuss their study involvement with the outcome assessor.

### Procedures

The FMI and HMI groups’ participants received the following five intervention components [9]: monitoring and management of metabolic and vascular risk factors, cognitive training and social activity, physical exercise, nutritional guidance, and motivational enhancement. The intervention period lasted for 24 weeks. The content of the five intervention components was the same between the FMI and HMI. Participants in the FMI group visited a facility three times a week to perform all intervention programs in a group or in individual sessions. Group sessions consisted of either 5 or 10 participants, depending on the size of the participating center. The HMI participants visited a facility once every 1-2 weeks to perform some programs in a group or in individual sessions and performed others at home. The detailed intervention is described in Table 5.

**Table 5:** Multidomain intervention.

**Intervention component**	**Facility-based multidomain intervention**	**Home-based multidomain intervention**
Monitoring and management of metabolic and vascular risk factors	Before the intervention, metabolic and vascular risk factors were evaluated through blood tests, alcohol and smoking habits, blood pressure, weight, body mass index, and waist circumference. At baseline and at week 12, participants met a study doctor who informed them of their risk factors; medications were prescribed when necessary. Additionally, participants received educational booklets corresponding to their risk factors and a booklet regarding lifestyle guidelines to prevent dementia. They met with a study nurse every 4 weeks for anthropometric measurements and to monitor their smoking and alcohol consumption.	
Cognitive training and social activity	Cognitive training was conducted, using a tablet-based application. Participants who struggled with using a tablet, were provided with workbooks. Cognitive training targets the cognitive domains of episodic memory, executive function, attention, working memory, calculation, and visuospatial function. Homework, a diary entry on a structured form, was assigned twice a week. Social activities were stimulated through numerous group meetings related to intervention components; additional social activities (e.g., theater, meeting friends, etc.) were conducted outside the facilities once a month.	
	Twice a week for 50 min in a group under the supervision of a trained health professional at a facility	During the first 2 months, the participants engaged in one group cognitive training session (each lasting 50 min) under the supervision of a trained health professional at a facility and one home-based cognitive training session per week. For the remainder of the 6-month intervention, they attended one group cognitive training session at a facility every two weeks. For the weeks that included the facility-based group session, participants performed one cognitive training session at home. In the weeks that did not include the facility-based group session, participants performed two cognitive training sessions at home.
Physical exercise	The physical exercise program consisted of aerobic, balance, flexibility, muscle-strengthening, and finger-and-toe exercises; exercises were provided three times a week for 60 min. Trained exercise professionals guided the group sessions at a facility; portable tools such as elastic bands, floor plates with numbers, and chairs were utilized. Every 2 months, exercise intensity was increased, and exercise content was changed.	
	Three times a week for 60 min in a group at a facility	During the first 2 months, the participants engaged in one group exercise session at a facility and two home-based exercise sessions per week. For the remainder of the 6-month intervention, they attended one group exercise session at a facility every two weeks. For the weeks that included the facility-based group exercise session, participants performed two exercise sessions at home. In the weeks that did not include the facility-based group exercise session, participants performed three exercise sessions at home. Participants exercised at home watching exercise videos on a tablet PC or following the instructions on a poster or booklet.
Nutritional guidance	The nutrition intervention was based on the Mediterranean-Dietary Approaches to Stop Hypertension Intervention for Neurodegenerative Delay (MIND) diet [39]. A licensed dietitian led the 30-min individual counseling sessions and 50-min group education sessions. In individual sessions, participants were educated on personalized diets to manage individual vascular risk factors. The group sessions provided practical exercises towards facilitating dietary changes; several cooking lessons provided advice on how to make meals with the recommended ingredients. The participants were monitored through a MIND diet checklist that they wrote every 2 weeks.	
	Three individual counseling sessions and seven group education sessions at a facility	Three individual counseling sessions and four group education sessions at a facility, and three home-based sessions using a workbook
Motivational enhancement	In 50-min group counseling sessions conducted by psychologists, participants’ motivation was strengthened, and they were educated regarding the importance of lifestyle changes for the prevention of dementia. In the FAMICO program, family members participated in strengthening participants’ motivation by creating cheering video messages. Encouraging pop-up video messages made by family members and self-assessment pop-up messages regarding participants’ dementia prevention activities were viewed by participants once a week before tablet-based cognitive training. Participants engaged in the workbook-type cognitive interventions received their encouraging video messages on their mobile phones; their self-assessments regarding dementia prevention activities were done on paper. Additionally, we strengthened participants’ motivation by sending dementia-related articles weekly as text messages, to their mobile phones.	
	Four group education sessions at a facility and weekly self-assessment regarding participants’ dementia prevention activities	Three group education sessions at a facility, one motivational enhancement program using a workbook at home, and weekly self-assessment regarding participants’ dementia prevention activities

At baseline, the participants in the control group met a study doctor, were prescribed medication when necessary, and received educational booklets corresponding to their risk factors and a booklet on lifestyle guidelines to prevent dementia. They received usual care during the study period and were informed that they could participate in the multidomain intervention program after the study ended.

### Outcomes

The primary outcome was feasibility measured through adherence, retention rates, and changes in the total scale index score of the RBANS with the normative data of Korean adults throughout the study [40]. The RBANS includes 12 subtests and evaluates five different cognitive domains: attention, language, visuoconstruction, immediate memory, and delayed memory. The index score (range 40-160) of each cognitive domain is separately scaled by age group to a scaled score mean of 100 with associated SD of 15. The total scale index score (range 40-160) is derived from the sum of five index scores, also with a normal mean of 100 and standard deviation of 15 [16].

Adherence to the intervention during sessions carried out at the facilities was assessed in real time. A tablet-based cognitive application was configured to allow administrators to view all participants’ data on the administration homepage. Study coordinators assessed adherence to tablet-based cognitive training home sessions on the administration homepage or by checking their cognition workbooks. They examined adherence to the exercise program’s home sessions by comparing the written self-reports with those recorded by a Fitbit smartwatch (Fitbit, Inc., San Francisco, CA, USA) on the participants’ wrists while they performed their daily activities. Participants wrote down the day and time when they performed the exercise program. We subsequently cross-checked their written self-report with the recorded Fitbit activity. Adherence to the nutritional and motivational home sessions was assessed by examining the participants’ homework. Furthermore, adherence to additional social activities (e.g., theater, meeting friends, etc.) was assessed by examining written self-reports. The retention rate was calculated as the percentage of participants in each group who did not discontinue the study.

The secondary outcomes were derived from measures that evaluated the effectiveness of the intervention’s components. The measurement tools included the following: MMSE [12], CDR-SB [14], Prospective Memory Test (PMT) modified from the Royal Prince Alfred PMT [10, 41], PRMQ for evaluating memory problems perceived by a proxy [19], cognitive complaint interview for evaluating subjective memory problems [42], GDS-15 [13], Bayer-ADL [15], and QOL-Alzheimer’s disease (QOL-AD) [43]. Participants’ physical functioning was assessed with the Global Physical Activity Questionnaire [44], SPPB [20], and K-NPFEP. The K-NPFEP measures grip power, sit-to-stand for 30 sec, walk in place for 2 min, bending the upper body forward, TUG test, quickly walking along a figure-eight track, and bioelectrical impedance analysis. Dietary intake was assessed with the Mini Nutritional Assessment [45] and NQ-E [17]. The NQ-E was developed by the Korean Nutritional Society to assess how often participants eat vegetables, fruit, beans, fish, milk, dairy products, eggs, water, fast food, pastries, and sweets [17]. Motivation was assessed by the SDI [18]. Changes in BP, body mass index, waist circumference, smoking, and alcohol consumption were evaluated. Blood tests were performed to assess folate, vitamin B12, 25-hydroxyvitamin D, homocysteine, and lipid and glucose levels.

The RBANS and PMT were assessed at baseline and within 4 weeks after the last intervention. Other secondary outcomes were assessed within 4 weeks before the intervention and within 4 weeks after the last intervention. Participants who withdrew prematurely were asked to complete all endpoint assessments at the point of early termination. Study coordinators evaluated the occurrences of AEs when participants visited a facility, when AEs took place, and at the end of the study.

### Exploratory outcomes of blood biomarkers

We performed exploratory studies to investigate the mechanism by which multidomain intervention works in the brain [9]. Changes in plasma total cortisol, a major hormonal byproduct of the HPA axis system [26], serum BDNF as a biomarker of neuroplasticity, NFL as a biomarker of neurodegeneration, and YKL-40 as a neuroinflammatory marker were investigated after the multidomain intervention Fasting blood samples were collected from all participants at approximately 9 o’clock in the morning in serum separator tubes (SSTs) and in K2EDTA tubes within 4 weeks before baseline and at the study endpoint. The SSTs were maintained at room temperature for 30 min. The SSTs and K2EDTA tubes were centrifuged for 10 min at 3000 rpm. In 0.5 mL aliquots, serum and plasma samples were stored in cryovial tubes at ≤ -70° C until analysis. Serum BDNF, NFL, and YKL-40 were measured via a quantitative sandwich enzyme-linked immunosorbent assay using DBD00 and DC3L10 (R&D Systems, Inc., Minneapolis, MN, USA) for BDNF and YKL-40, respectively, and LS-F6701 (LifeSpan BioSciences, Inc., Seattle, WA, USA) for NFL, according to the manufacturers’ instructions. Plasma total cortisol levels were determined by liquid chromatography-selected reaction monitoring quantification using a QTrap5500 mass spectrometer (ABSciex, Foster City, CA, USA). The detailed method is described in the Supplementary Method.

### Statistical analysis

Since the purpose of this feasibility trial was to estimate retention and adherence rates, the sample size was calculated using the 95% CI for the rates. Based on the recommendations of sample size calculations for feasibility studies from the National Institute for Health Research [46], we could estimate the retention and adherence rates of 75% when the sample size was 50 and within a 95% CI of ± 12%. Therefore, the required sample size was determined to be 150, with 50 participants in each group.

The adherence rate was calculated by adding the number of sessions completed and dividing by the number of interventions assigned to each intervention component: vascular and metabolic risk factor management (6 sessions), cognitive training (48 sessions), additional social activity (5 sessions), physical exercise (72 sessions), nutrition education (10 sessions), and motivational enhancement (4 education sessions and 24 self-assessments of dementia prevention activities). The total adherence rate was calculated by adding the number of sessions completed across all intervention components and dividing by the total number of interventions (169 sessions) assigned without modifying the weight of each intervention component. Regarding the participants who dropped out of the study, adherence rates were calculated until the dropout point. The chi-square test was used to compare the retention rates between the intervention and control groups.

The RBANS, secondary outcomes, and exploratory blood biomarkers were analyzed using a mITT population. This population was defined as all participants who received baseline evaluation and at least one postbaseline assessment and participated at least once in the intervention program. We used a chi-square test for categorical variables and a one-way analysis of variance for continuous variables to compare baseline characteristics between the groups. Analysis of covariance (ANCOVA), with a baseline score as a covariate, was used to compare changes from baseline to the study endpoint in the RBANS, secondary outcomes, and exploratory blood biomarkers between each intervention group and the control group. *P* values in the analysis regarding changes in the RBANS total scale index score were adjusted for multiple comparisons by the Bonferroni correction, accounting for the fact that the two intervention groups were compared with one control group.

The safety analysis was conducted on participants who underwent at least one safety evaluation after baseline and participated at least once in the intervention program. The chi-square test was used to compare the prevalence of AEs between the intervention and control groups. Statistical analyses were performed using SPSS 26.0 (SPSS, Chicago, IL, USA). *P* < 0.05 was considered significant.

## Supplementary Material

Supplementary Materials

Supplementary Tables
