# Supplementary material for: Facility-based and home-based multidomain interventions including cognitive training, exercise, diet, vascular risk management, and motivation for older adults: a randomized controlled feasibility trial
Source: Aging (Albany NY). 2021 Jun 18;13(12):15898–916. doi: 10.18632/aging.203213 (PMC8266338; doi:10.18632/aging.203213)
Supplement: Supplementary Materials [file aging-13-203213-s001.pdf]

SUPPLEMENTARY MATERIALS

Supplementary Method

Detailed method to measure plasma cortisol level

Chemicals

Cortisol and a deuterated internal standard, cortisol-d4, as an internal standard were purchased from Cerilliant (Round Rock, TX, USA). Methyl tert-butyl ether (MTBE), HPLC-grade water, acetonitrile, methanol, and formic acid (≥ 98%) were purchased from Merck (Darmstadt, Germany).

Sample preparation

Stock solutions of cortisol and cortisol-d4 were prepared in methanol at concentrations of 1 mg/mL and 100 µg/mL, respectively. A 150 µL aliquot of plasma samples was prepared by adding 50 µL of the deuterated internal standard, cortisol-d4 (100 ng/mL). The samples were vortex-mixed for 10 s, followed by adding 2 mL of MTBE for phase separation, vortexing gently for 15 min. After centrifuging at 4000 rpm for 5 min, the organic layer was collected and evaporated to dryness under nitrogen gas at 60° C. The extract was reconstituted with 100 µL of acetonitrile, and 10 µL of the analyte was used for further liquid chromatography-selected reaction monitoring (LC-SRM) measurements.

SRM optimization

10 µg/mL of cortisol and cortisol-d4 was prepared and directly infused into a QTrap5500 hybrid linear ion-trap triple quadrupole mass spectrometer (MS) (ABSciex, Foster City, CA, USA), equipped with TurboSpray source (ABSciex, Foster City, CA, USA). MS was optimized in the positive mode with the following instrumental parameters: curtain gas at 20 psi, nebulizer at 30 psi, ion spray voltage of 5000 V, temperature at 150° C, scan mass range of 50-500 m/z, scan rate at 200 Da/s, resolution at 0.7 Da (unit resolution), injection flow at 7 µL/min with a syringe (i.d., 4.6 mm). The most intense transitions were selected for further LC-SRM quantification: 363.2 → 121.1 for cortisol and 367.1 → 121.1 for cortisol-d4. SRM parameters such as declustering potential, collisional energy, and collisional cell exit potential were then optimized at 71 V, 60 V, and 10 V for cortisol, and 86 V, 27 V, and 8 V for cortisol-d4, respectively.

LC-SRM quantification

SRM quantitation was performed on QTrap5500 equipped with an Agilent 1200 series (Palo Alto, CA, USA). The samples were injected and separated with a Gemini C18 column (2.0 mm, i.d., x 50mm, l., 5 µm particle size) (Phenomenex, USA). The mobile phase consisted of HPLC grad water with 0.5% formic acid and acetonitrile with 0.5% formic acid (10:90, v/v) and was delivered at a flow rate of 0.35 mL/min. The temperatures of autosampler and column oven were 8° C and 35° C, respectively. Analyst software (version 1.5.1, Applied Biosystems/MDS Sciex, Foster City, CA, USA) was used to monitor SRM data and to elucidate optimized SRM parameters.
