# Supplementary material for: Facility-based and home-based multidomain interventions including cognitive training, exercise, diet, vascular risk management, and motivation for older adults: a randomized controlled feasibility trial
Source: Aging (Albany NY). 2021 Jun 18;13(12):15898–916. doi: 10.18632/aging.203213 (PMC8266338; doi:10.18632/aging.203213)
Supplement: Supplementary Tables [file aging-13-203213-s002.pdf]

SUPPLEMENTARY TABLES

Supplementary Table 1. Baseline characteristics of all randomized participants.

	FMI group (n = 51)	HMI group (n = 51)	Control group (n = 50)	*P**
**Demographic characteristics**				
Age at baseline visit, years	71.6 (4.7)	71.0 (4.9)	70.6 (4.8)	0.58
Number of women	37 (72.5%)	37 (72.5%)	39 (78.0%)	0.77
Education, years	10.2 (4.8)	9.9 (5.2)	10.5 (4.8)	0.82
**Medical history**				
Hypertension	24 (47.1%)	28 (54.9%)	29 (58.0%)	0.52
Diabetes mellitus	11 (21.6%)	13 (25.5%)	18 (36.0%)	0.25
Dyslipidemia	30 (58.8%)	33 (64.7%)	33 (66.0%)	0.73
Cardiac disease	3 (5.9%)	2 (3.9%)	3 (6.0%)	0.87
History of stroke	3 (5.9%)	3 (5.9%)	5 (10.0%)	0.66
Mild cognitive impairment	17 (33.3%)	14 (27.5%)	12 (24.0%)	0.57
**Vascular factors**				
Systolic blood pressure, mmHg	127.1 (16.1)	127.0 (13.0)	130.7 (16.3)	0.38
Diastolic blood pressure, mmHg	73.3 (10.6)	74.5 (10.1)	73.4 (9.0)	0.82
Total cholesterol, mg/dL	183.0 (40.2)	189.7 (35.7)	176.7 (44..0)	0.27
LDL-cholesterol, mg/dL	102.7 (37.5)	111.0 (32.9)	99.1 (35.9)	0.23
Triglyceride, mg/dL	137.9 (71.4)	142.3 (78.0)	150.5 (109.4)	0.77
HDL-cholesterol, mg/dL	53.6 (12.3)	53.7 (14.5)	52.2 (12.8)	0.84
Fating plasma glucose, mg/dL	105.2 (32.2)	110.0 (33.4)	109.5 (38.8)	0.75
Body Mass Index, kg/m^2	23.8 (2.2)	24.3 (3.1)	24.7 (3.0)	0.27
Abdominal circumference, cm	82.6 (7.4)	84.5 (8.8)	84.9 (8.2)	0.33
**Lifestyle factors**				
Current smokers	2 (3.9%)	1 (2.0%)	1 (2.0%)	0.78
At-risk alcohol drinking†	7 (13.7%)	6 (11.8%)	2 (4.0%)	0.22
Physical activity, MET × min per week	2449 (2719)	2773 (4594)	2302 (2655)	0.78
**Cognition**				
Mini-Mental State Examination	28.1 (1.7)	27.9 (1.9)	27.2 (2.4)	0.08
RBANS total scale index score	101.2 (19.0)	100.5 (17.8)	99.8 (19.8)	0.94
Clinical Dementia Rating-Sum of Boxes	0.48 (0.57)	0.51 (0.61)	0.39 (0.42)	0.52
Geriatric Depression Scale-15 items	4.5 (4.0)	4.2 (4.2)	4.0 (3.4)	0.75

Values are shown as mean (SD) or number (%). FMI, facility-based multidomain intervention; HMI, home-based multidomain intervention; LDL, low-density lipoprotein; HDL, high-density lipoprotein; MET, metabolic equivalents; RBANS, Repeatable Battery for the Assessment of Neuropsychological Status. *Chi-square test for categorical variables and analysis of variance for continuous variables. †Four drinks or more during a day, or more than seven drinks per week.

Supplementary Table 2. Mean changes in the secondary outcome measures from baseline to the study endpoint.

	Baseline scores			Changes from baseline to study end			*P**	
	FMI (n = 48)	HMI (n = 50)	Control (n = 42)	FMI (n = 48)	HMI (n = 50)	Control (n = 42)	FMI vs. Control	HMI vs. Control
**Physical fitness**								
Left grip power, kg	24.0 (8.7)	23.9 (7.4)	20.7 (5.5)	0.63 (2.89)	1.30 (3.19)	0.98 (2.61)	0.98	0.48
Right grip power, kg	25.2 (9.3)	25.5 (7.4)	23.6 (5.8)	0.46 (2.84)	0.99 (3.24)	0.36 (2.61)	0.57	0.27
Sit-to-stand for 30 sec	17.7 (5.9)	18.0 (5.0)	17.4 (6.7)	4.57 (6.51)	3.73 (5.42)	2.13 (8.06)	0.08	0.14
Bending upper body forward, cm	13.0 (9.2)	11.4 (11.4)	10.6 (10.6)	0.96 (5.26)	-0.21 (5.01)	-0.28 (5.18)	0.06	0.84
Walk in place for 2 min	99.5 (27.3)	107.9 (20.4)	97.8 (27.5)	16.15 (28.28)	9.75 (21.87)	0.54 (23.22)	**0.001**	**<0.001**
Fast walking along a figure-eight track, sec	27.3 (5.5)	28.6 (6.0)	27.8 (5.4)	-0.40 (5.95)	-2.42 (5.95)	2.23 (7.54)	**0.01**	**<0.001**
**Vascular factors**								
Body mass index, kg/m^2	23.8 (2.1)	24.3 (3.1)	25.1 (2.8)	-0.18 (0.98)	0.03 (0.65)	0.08 (0.75)	0.05	0.73
Waist circumference, cm	82.7 (7.1)	84.5 (8.9)	85.6 (8.4)	-1.07 (4.07)	-1.97 (4.28)	-0.53 (4.63)	0.18	0.10
Binge drinking per month	0.6 (2.9)	0.7 (2.9)	0.1 (0.3)	-0.33 (1.48)	-0.28 (0.95)	-0.05 (0.31)	0.58	0.53
Total cholesterol, mg/dL	184.9 (39.7)	190.6 (35.5)	174.3 (41.2)	-7.25 (38.53)	-6.69 (32.32)	-0.51 (36.27)	0.89	0.85
LDL-cholesterol, mg/dL	103.9 (37.5)	111.7 (32.9)	95.8 (32.8)	-5.54 (32.27)	-4.53 (31.16)	0.44 (28.08)	0.70	0.67
HDL-cholesterol, mg/dL	53.9 (12.6)	53.8 (14.7)	52.5 (13.5)	3.44 (7.56)	2.14 (8.34)	3.44 (9.16)	0.89	0.60
Triglyceride, mg/dL	139.5 (72.9)	143.1 (78.6)	154.0 (117.9)	-20.92 (69.87)	-13.14 (73.04)	-26.51 (120.96)	0.46	0.88
LP(a), mg/dL	16.5 (19.1)	23.4 (21.3)	25.4 (27.8)	2.47 (24.61)	-0.08 (29.33)	-2.10 (21.33)	0.90	0.75
Fasting glucose, mg/dL	102.7 (29.9)	110.2 (33.7)	110.0 (39.9)	-10.85 (25.09)	-14.04 (30.91)	-7.13 (43.00)	0.07	0.17
HbA1c, %	5.87 (0.74)	5.90 (0.73)	6.11 (0.77)	-0.09 (0.35)	-0.02 (0.63)	-0.09 (0.35)	0.48	0.86
C-peptide, ng/mL	2.02 (1.59)	2.52 (2.11)	2.04 (1.60)	-0.42 (1.36)	-0.50 (1.83)	0.17 (1.75)	**0.04**	0.16
**Nutritional factors**								
Vitamin B12, pg/mL	687.8 (305.2)	717.2 (326.2)	771.2 (336.1)	460.11 (2913.12)	-47.13 (415.70)	635.41 (3061.06)	0.97	0.16
Folate, ng/mL	16.1 (16.7)	17.5 (14.2)	15.2 (10.7)	-1.87 (17.11)	2.48 (18.96)	4.16 (17.25)	0.13	0.81
25-OH vitamin D, ng/mL	21.0 (12.0)	23.2 (11.1)	21.6 (11.2)	2.47 (8.74)	5.39 (11.93)	4.86 (9.69)	0.22	0.67
Homocysteine, µmol/L	12.5 (2.4)	12.9 (2.7)	13.0 (3.6)	1.30 (2.50)	1.48 (3.73)	0.21 (3.40)	0.15	0.10
**Blood biomarkers**								
BDNF, ng/mL	25.48 (10.40)	27.54 (11.44)	30.27 (13.42)	11.83 (20.06)	0.67 (20.61)	-1.62 (19.01)	**0.02**	0.33
Cortisol, ng/mL	97.56 (211.45)	86.47 (165.10)	110.32 (307.08)	-5.29 (154.01)	-15.29 (172.09)	54.18 (136.01)	**0.049**	**0.03**
Neurofilament light chain, pg/mL	16.11 (9.56)	13.85 (7.95)	14.51 (9.06)	-4.21 (7.65)	-0.62 (8.74)	-1.98 (9.77)	0.29	0.55
YKL-40, ng/mL	90.24 (86.98)	75.66 (66.60)	67.31 (66.79)	-2.19 (65.33)	-12.76 (68.58)	4.62 (57.49)	0.90	0.26

Values are shown as the mean (SD). FMI, facility-based multidomain intervention; HMI, home-based multidomain intervention; LDL, low-density lipoprotein; HDL, high-density lipoprotein; LP, lipoprotein; HbA1c, hemoglobin A1c; BDNF, brain-derived neurotrophic factor. *Analysis of covariance with each baseline score as a covariate.
